# Rotational Three-dimensional OCTA: a Notable New Imaging Tool to Characterize Type 3 Macular Neovascularization

**DOI:** 10.1038/s41598-019-53307-x

**Published:** 2019-11-19

**Authors:** Enrico Borrelli, Riccardo Sacconi, Gerd Klose, Luis de Sisternes, Francesco Bandello, Giuseppe Querques

**Affiliations:** 1 Department of Ophthalmology, University Vita-Salute, IRCCS Ospedale San Raffaele, Milan, Italy; 2 Carl Zeiss Meditec, Inc., Tokyo, Japan; 3 Research and Development, Carl Zeiss Meditec, Inc., Dublin, CA USA

**Keywords:** Retinal diseases, Diagnostic markers

## Abstract

This study explored whether rotational three-dimensional (3D) visualization of optical coherence tomography angiography (OCTA) volume data may yield valuable information regarding type 3 macular neovascularization (MNV). In this retrospective, cross-sectional study, we collected data from 15 eyes (13 patients) with treatment-naïve type 3 MNV in their post-nascent stage and age-related macular degeneration (AMD). Subjects were imaged with the SS-OCT system (PLEX Elite 9000, Carl Zeiss Meditec Inc., Dublin, CA, USA). The OCTA volume data were processed with a prototype volume projection removal algorithm and then analyzed using volumetric visualization techniques in order to obtain a 3D visualization of the region occupied by type 3 MNV. The two-dimensional and three-dimensional OCTA images were investigated. Mean ± SD age was 75.1 ± 7.4 years. BCVA was 0.42 ± 0.21 LogMAR in the study eyes. Considering the cohort of analyzed eyes, on rotational 3D OCTA images, a total of 35 neovascular lesions (vs 22 lesions detected on 2D OCTA images) rising from the deep vascular complex and variably spanning the outer retinal layers and eventually reaching the RPE/sub-RPE space were detected. Nine of 35 lesions had a saccular shape, while the remaining cases had a filiform shape. On rotational 3D OCTA images, these lesions were inclined on the three planes, instead of perpendicular to the RPE/Bruch’s membrane. In conclusion, this study used an algorithm to obtain rotational three-dimensional visualization of type 3 MNV. This approach seems to increase the detection rate for these lesions and to be useful to offer new insight into type 3 MNV.

## Introduction

Type 3 macular neovascularization (MNV) represents a distinct form of intraretinal neovascularization that is usually associated with age-related macular degeneration (AMD). This type of MNV was first identified by Hartnett *et al*.^1^ in 1992, who termed this lesion a “retinal vascular abnormality”. In a subsequent paper^2^, they suggested to slightly change the term of this lesion in “retinal vascular anomalous complex”. Later, Yannuzzi *et al*.^3^ coined the term retinal angiomatous proliferation (RAP) and, more recently, this entity was renamed type 3 MNV^4–6^.

Optical coherence tomography angiography (OCTA) is a relatively novel imaging technique that produces volumetric angiographic images by performing repeated OCT acquisitions in the same tissue location within a short time to detect scattering differences that relate to motion produced by blood flow in the retinal and choroidal microvasculature^7,8^. OCTA studies have significantly expanded our knowledge on type 3 MNV. Importantly, using structural OCT and OCTA, previous important studies displayed that type 3 MNV arises from the deep vascular complex (DVC) and progresses downward toward the retinal pigment epithelium (RPE) leading to exudation and pigment epithelial detachment (PED)^9–15^. Importantly, OCTA may be also useful in the identification of precursor lesions with flow (nascent type 3 MNV), corresponding to intraretinal hyperreflective foci identified on structural OCT, that may precede more mature type 3 lesions associated with more significant exudation (post-nascent stage of type 3 MNV)^16,17^.

While OCT is considered a cross-sectional imaging modality, OCTA images are mainly displayed with *en face* visualization. The *en face* images may be obtained by segmenting the volumetric OCTA scan at specific depths. These volume sections defined by two topographic surfaces are called slabs. Using this strategy, the flow data within any slab are summed or projected into a two-dimensional *en face* image^7^. However, this visualization may not be appropriate for exhibiting lesions that principally develop vertically (e.g. type 3 MNV). For this reason, cross-sectional OCTA scans may be helpful for studying these lesions. Flow information is typically depicted within cross-sectional OCT data by a pseudocolor overlay on the grayscale structural OCT image. However, the latter strategy is limited to the visualization of two-dimensional (2D) images which do not allow for the appreciation of the whole type 3 lesion, which could be oriented on the three dimensions.

Therefore, while the 2D visualization remains the gold standard for evaluating OCTA images, numerous limitations exist, primarily for displaying anatomical entities where both their *en face* extent and depth information is important to better understand their status and behavior, such as type 3 MNV. These shortcomings may be partially addressed with the development of three-dimensional (3D) illustrations, assuming that the acquired OCTA volume enables 3D visualization of the retinal and choroidal microvasculature. This approach has been previously successfully employed by Spaide to describe a range of retinal vascular abnormalities^18,19^. Of note, these 3D visualizations may be rotated on the 3 axes and this may further reduce limitations by overlapping anatomy and vessel foreshortening, as demonstrated for other imaging modalities (e.g. cerebral angiograms)^20^.

In this study we thus explored this 3D representation in eyes with treatment-naïve post-nascent stage type 3 MNV and AMD. To obtain this visualization we employed a novel process with a sophisticated algorithm to reduce volumetric projection artifact. Our aim was to understand whether the three-dimensional images generated by this technique yield valuable information regarding the configuration of type 3 lesions.

## Methods

### Study participants

This study is a retrospective, cross-sectional study. The authors in this study identified patients with treatment-naïve post-nascent stage type 3 MNV as determined by clinical examination, structural OCT and OCTA evaluation^5,6,9–12,16,17^. Fluorescein and indocyanine green angiographies were performed to enhance diagnosis. The study was approved by the San Raffaele Institutional Review Board and adhered to the tenets of the Declaration of Helsinki and Health Insurance Portability and Accountability Act. Written informed consent was obtained from all subjects.

Exclusion criteria included (i) previous ocular surgery or history of anti-vascular endothelial growth factor (VEGF) therapy; (ii) any maculopathy secondary to causes other than AMD (including presence of an epiretinal membrane or vitreomacular traction syndrome). Furthermore, we excluded poor quality images with a signal strength index lower than 6 (a measurement in a scale 0–10 indicating the level of retinal tissue signal with respect to the noise or background level in OCT data), as recommended by manufacturers.

All patients underwent a complete ophthalmological examination including best-corrected visual acuity (BCVA) assessment, slit lamp bio-microscopy and fundus examination by an experienced retina specialist.

### OCTA imaging

Patients underwent SS-OCTA imaging using the PLEX Elite 9000 device (Carl Zeiss Meditec Inc., Dublin, CA, USA) which uses a swept laser source with a central wavelength of 1050 nm (1000–1100 nm full bandwidth) and operates at 100,000 A-scans per second. This instrument employs a full-width at half-maximum (FWHM) axial resolution of approximately 5 μm in tissue, and a lateral resolution at the retinal surface estimated at approximately 14 μm. OCTA imaging of the macula included a 3 × 3-mm field of view area centered on the fovea (300 A-scans × 300 B-scans).

### Image processing

To mitigate the influence of the projection effect on the three-dimensional visualization of the type 3 lesions, we used a prototype version of a volume projection removal algorithm. This algorithm is aimed at generating OCTA volumes without (or at least with less) projection artifacts. Different from slab-based artifact removal solutions, this algorithm proposes a solution to resolve artifacts within a volume instead of a single slab. In brief, the basic principle of this algorithm (available on the ARI network by Zeiss - https://arinetworkhub.com) consists on a set of serial consecutive steps in which a corrected volume is updated. At each step, a thin (small axial thickness) portion of the OCTA volume (target subvolume) is partially corrected and stored using a previously corrected portion of the OCTA volume located at inner positions. The target subvolume considered at each step is located at increasing depths in an overlapping manner. Using this process, each voxel within the OCTA volume is corrected in several consecutive steps.

**Figure 1:**
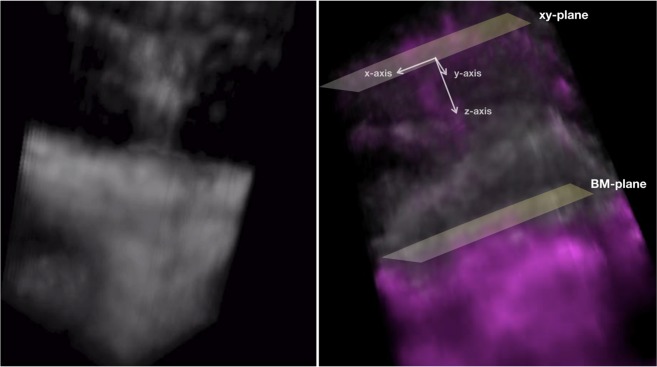
Frames obtained from rotational 3D visualizations of a type 3 neovascularization. The 3D OCTA visualization (**left image**) demonstrates the presence of an intraretinal lesion originating from the deep vascular complex and moving toward the outer retinal layers. The merged 3D structural OCT (white signal) and OCTA (magenta signal) visualization (**right image**) allowed to investigate correlations between flow and structural data. The orientation of type 3 lesions was assessed in relation with the three axes of the three-dimensional Cartesian coordinate system, assuming that the xy-plane passes through the type 3 lesion’s origin and is parallel to the Bruch’s membrane (BM) plane.

The obtained corrected OCTA volume scans were thus exported as uncompressed 8-bit raw-data file and imported in ImageJ software version 1.50 (National Institutes of Health, Bethesda, MD; available at http://rsb.info.nih.gov/ij/index.html)^21^ and re-oriented in space so that the first dimension correspond to the axial direction with increased pixel number as increasing depth, and the second and third dimensions correspond to the horizontal and vertical directions, respectively. Finally, the “3D Viewer” plugin (downloadable on http://3dviewer.neurofly.de) was carried out to obtain the 3D visualization of type 3 lesions (Fig. 1).

In order to study the relationship between structural OCT and OCTA-detected flow, structural OCT volume and OCTA volume data were merged. Therefore, the “3D Viewer” plugin was carried out to obtain a second 3D visualization in which structural OCT and OCTA information had different colors (Fig. 1). The latter visualization was used to correlate OCTA signal with structural OCT information. In detail, the position and orientation of type 3 lesions in relation to retinal structures, RPE and Bruch’s membrane was qualitatively investigated. The orientation of type 3 lesions was assessed in relation with the three axes of the three-dimensional Cartesian coordinate system, assuming that the xy-plane passes through the type 3 lesion’s origin and is parallel to the Bruch’s membrane plane (Fig. 1).

### Grading and statistical analysis

The two-dimensional and three-dimensional OCTA findings were carefully examined by two experienced graders (EB and GQ). Each grader performed this assessment separately. Graders later met to compare level of agreement, and disagreements were resolved by further discussion and open adjudication to yield a single assessment for each case. The analysis included descriptive statistics (using Microsoft Office Excel software; version 14.0, 2010, Redmond, WA) for demographics and main clinical data, and qualitative descriptions of the imaging characteristics.

## Results

A total of 15 treatment-naïve eyes with type 3 MNV from 13 AMD patients were included in the analysis. Mean ± SD age was 76.2 ± 7.3 years [range 70–91 years]. Mena BCVA was approximately 20/40 Snellen in the study eyes. The two graders agreed in their observations in 15 out of 15 eyes.

In all study eyes, type 3 MNV overlaid a variable sized PED. The presence of a fibrovascular PED featuring the co-presence of a type 1 MNV was evident in 7 out of 15 eyes on ICGA and/or OCTA images (Table 1). On late-phase ICGA images, type 3 lesions were identified as leaking hotspots. This assessment revealed the presence of 19 lesions [mean ± SD was 1.3 ± 0.6; range was 1.0–3.0].

**Table 1:** Characteristics of analyzed type 3 lesions.

**Enrolled eyes, n**	15
**Presence of associated T1 MNV, n (%)**	7 (46.7%)
**T3 MNV identified with 2D OCTA, n**	22
**T3 MNV identified with 3D OCTA, n**	35
**Shape**	
- Saccular, n (%)	9 (25.7%)
- Filiform, n (%)	26 (74.3%)

**n:** number; **T1 MNV:** type 1 macular neovascular lesion; **T3 MNV:** type 3 macular neovascular lesion; **OCTA:** optical coherence tomography angiography.

**Figure 2:**
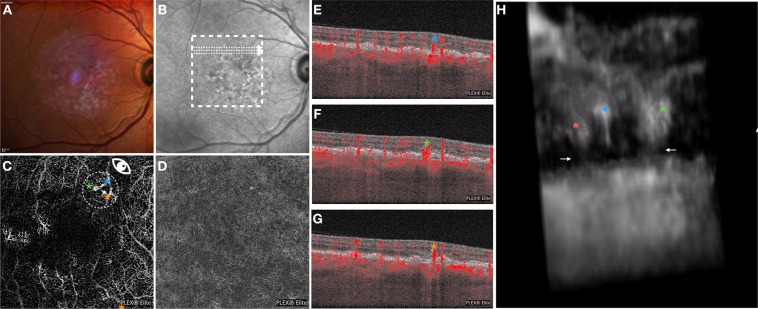
Multimodal imaging of the RE of a 80-year-old woman diagnosed with type 3 neovascularization. Multicolor (**A**) and infrared (**B**) images show areas of RPE alteration and mottling in the macula. *En face* OCTA images segmented at the outer retina (**C**) and RPE-RPE fit (**D**) slabs show 3 tuft-shaped high-flow lesions (highlighted with 3 asterisks) and absence of sub-RPE neovascularizations, respectively. The white arrows on the infrared reflectance image show the location and direction of the OCTA B-scans (**E**–**G**) that display 3 high-flow vessels descending toward the outer retinal layers. The 3D OCTA visualization (image **H** is the frame obtained by visualizing the circular region of interest highlighted on image **C** and visualized from the angle marked with the white eye) displayed three distinct intraretinal lesions. Two lesions had a saccular shape (orange and green asterisks), while the other lesion (blue asterisk) had a filiform shape. The saccular-shaped lesions seem to be in contact with the choroid through small caliber vessels (white arrows).

**Figure 3:**
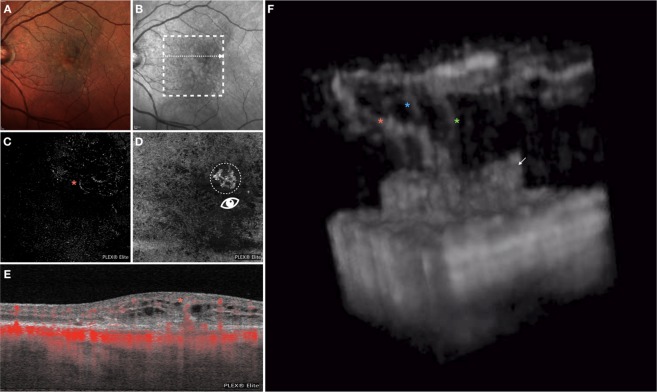
Multimodal imaging of the LE of an 82-year-old woman diagnosed with type 3 neovascularization. Multicolor (**A**) and infrared (**B**) images show areas of RPE alteration and mottling in the macula. *En face* OCTA images segmented at the outer retina (**C**) and RPE-RPE fit (**D**) slabs show one tuft-shaped high-flow lesion (highlighted with an orange asterisk) and presence of a sub-RPE neovascularization, respectively. The white arrow on the infrared reflectance image shows the location and direction of the OCTA B-scan (**E**), this displaying an high-flow vessel originating from the deep vascular complex and reaching the sub-RPE space. The 3D OCTA visualization (image **F** is the frame obtained by visualizing the circular region of interest highlighted on image **D** and visualized from the angle marked with the white eye) revealed the presence of three distinct intraretinal lesions with a filiform shape (marked with 3 asterisks), which merge in the outer retinal layers, and one sub-RPE neovascularization (white arrow).

**Figure 4:**
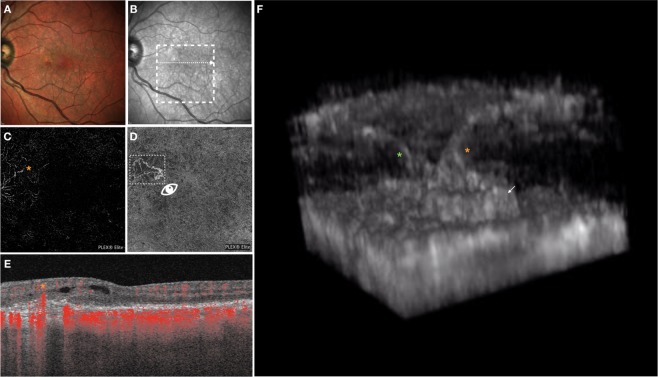
Multimodal imaging of the LE of a 70-year-old woman diagnosed with type 3 neovascularization. Multicolor (**A**) and infrared (**B**) images show areas of RPE alteration and mottling in the macula. On the *en face* OCTA image segmented at the outer retina (**C**) a tuft-shaped high-flow lesion was displayed (orange asterisk), while the RPE-RPE fit (**D**) slab shows the presence of a sub-RPE neovascularization. On the OCTA B-scan (**E**), whose location and direction is shown with a white arrow on the infrared reflectance image, an high-flow lesion originating from the deep vascular complex and reaching the sub-RPE space was displayed. The 3D OCTA visualization (image **F** is the frame obtained by visualizing the square region of interest highlighted on image **D** and visualized from the angle marked with the white eye) revealed the presence of two distinct intraretinal lesions with a filiform shape (marked with 2 asterisks), that reach the sub-RPE space, where a neovascularization is located (white arrow).

On 2D OCTA images, all eyes showed at least 1 lesion characterized by a retinal–retinal anastomosis emerging from the DVC and forming a clear tuft-shaped high-flow network in the outer retinal layers and finally abutting in the sub-RPE space (Figs 2–4; Video 1). On 2D images, 22 type 3 lesions were displayed in the whole cohort of 15 eyes [mean ± SD was 1.4 ± 0.9; range was 1.0–4.0] (Table 1).

Considering the whole cohort of analyzed eyes, on rotational 3D OCTA images, a total of 35 neovascular lesions rising from the DVC and reaching the outer retinal layers were detected [mean ± SD was 2.4 ± 1.1 for each patient; range was 1.0–5.0] (Figs 3, 4; Video 2). Nine of 35 lesions had a saccular shape, while the remaining cases had a filiform shape. On rotational 3D OCTA images, these lesions were inclined on the three planes (Table 1, Fig. 1).

### Case presentation

#### Case 1

A 75-year-old woman with history of neovascular AMD in the left eye (LE) presented with sudden vision loss on her right eye (RE). Best corrected visual acuity was 20/30 in the RE and 20/100 in the LE. Structural optical coherence tomography revealed a hyperreflective intraretinal complex, emerging from the DVC, apparently connected with the sub-RPE space, with associated intraretinal exudation. Two-dimensional OCTA images of the RE displayed three tuft-shaped high-flow lesions (highlighted with three different asterisks in Fig. 2), which moved toward the outer retinal layers and abutted into the sub-RPE space, as shown on the B-scan OCTA images. The RPE-RPE fit segmentation revealed absence of a sub-RPE neovascular lesion. The 3D OCTA visualization displayed three distinct intraretinal lesions. These lesions emerged from the DVC and moved toward the sub-RPE space. Two of three lesions were characterized by a saccular shape and seemed to be in close contact with the choroid through small caliber vessels, while the third lesion had a filiform shape and did not show any evident connection with the choroid (Fig. 2, Video 1).

#### Case 2

An 82-year-old woman with a diagnosis of bilateral nonexudative AMD presented for metamorphopsia and vision loss in her LE. Best corrected visual acuity was 20/20 in the RE and 20/40 in the LE. Multimodal imaging revealed the presence of a type 3 lesion with associated intraretinal exudation. Two-dimensional OCTA displayed the presence of an intraretinal complex emerging from the DVC and apparently connected with the sub-RPE space. Of note, the RPE-RPE fit slab demonstrated the presence of a sub-RPE type 1 MNV. The 3D OCTA visualization displayed three distinct intraretinal lesions with a filiform shape emerging from the DVC. These three lesions are oblique on the three planes along their route toward the outer retinal layers, where they appear to connect with each other to shape a larger vascular trunk. The latter vessel appears to be connected with the sub-RPE space, where a type 1 MNV is evident (Fig. 3; Video 1).

#### Case 3

A 70-year-old woman with a diagnosis of bilateral nonexudative AMD presented for metamorphopsia and vision loss in her LE. Best corrected visual acuity was 20/20 in the RE and 20/40 in the LE. A diagnosis of type 3 MNV with associated intraretinal exudation was made. Two-dimensional OCTA displayed the presence of an intraretinal complex emerging from the DVC and apparently connected with the sub-RPE space, where a sub-RPE type 1 MNV was displayed. The 3D OCTA visualization displayed two distinct filiform-shaped intraretinal lesions which emerge from the DVC and move obliquely toward the sub-RPE space, where appear to be connected with the type 1 MNV (Fig. 4, Video 1).

**Figure 5:**
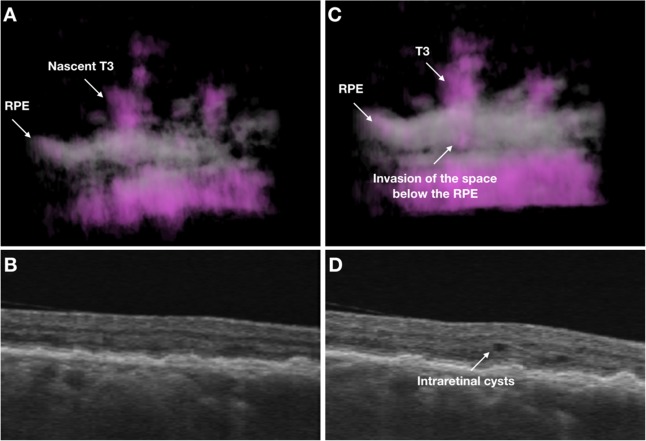
Multimodal imaging of the RE of a 74-year-old woman diagnosed with type 3 neovascularization. At baseline, the rotational 3D OCTA image (**A**) shows the presence of a nascent T3 neovascularization which seems to minimally invade the sub-RPE space. At this stage, the T3 MNV does not cause exudation, as displayed on the correspondent structural OCT B-scan (**B**). At the 1-month follow-up visit, the 3D OCTA visualization (**C**) demonstrates that the T3 MNV more significantly invades the sub-RPE space and this is associated with the presence of intraretinal fluid (**D**).

In a case with baseline and follow-up visits (Fig. 5, Video 2), at baseline the rotational 3D OCTA image displayed the presence of a nascent T3 neovascularization which seems to minimally invade the sub-RPE space. At this stage, the structural OCT demonstrated that this nascent T3 MNV does not cause exudation. At the 1-month follow-up visit, the 3D OCTA visualization demonstrated that the T3 MNV more significantly invades the sub-RPE space and this is associated with the presence of intraretinal fluid.

## Discussion

In this retrospective, cross-sectional study we applied a novel algorithm to volumetric OCTA data in order to obtain rotational three-dimensional visualizations of treatment-naïve type 3 MNV. Overall, we observed that this approach may be effective to display type 3 lesions and may offer new important insight into the pathogenesis and characterization of these lesions.

Over the last years, various authors have sought to clarify the origin and pathogenesis of type 3 MNV using both histopathologic and imaging studies. Histopathologic characteristics of type 3 lesions have been extensively studied in postmortem analyses on AMD eyes^22–25^. These studies demonstrated the presence of retinal vascular elements with collagenous material and Müller cell processes implanting into thick sub-RPE basal laminar deposit^22–25^. However, there was no histopathological evidence of any connection between choroidal vessels and intraretinal type 3 MNV^26,27^. Using structural OCT and OCTA, it was apparent that type 3 lesions are localized at the crest or apex of the PED after originating from the DVC^9–14,16,28,29^. Importantly, OCTA granted a trustworthy description of the microvascular morphology of type 3 MNV^13,14,16,28–30^. In detail, two-dimensional OCTA images illustrated type 3 MNV as lesions with a distinct high flow, tuft-like capillary network. Finally, OCTA evaluation of the CC in eyes with type 3 lesion demonstrated that flow abnormalities (presumably CC ischemia) may drive the development of type 3 MNV^31^. In our study cohort, the 2D OCTA images confirm findings from previous studies as we showed that treatment-naïve type 3 lesions emerge as retinal–retinal anastomosis from the DVC and connect with the sub-RPE space at the time of clinically detectable exudation.

We add to the literature by reporting the rotational 3D visualizations of treatment-naïve type 3 MNV. This approach was revealed to be useful for the identification and characterization of these lesions.

This three-dimensional approach allowed to recognize more lesions emerging from the DVC, as compared with the standard 2D visualization (35 vs 22 lesions identified, respectively). Taking into consideration that conventional *en face* and B-scan OCTA images may be limited by overlapping anatomy and vessel foreshortening, our results suggest that 3D images may be more appropriate to identify type 3 lesions by resolving these limitations and this visualization may be thus characterized by a higher detection rate. Furthermore, assuming that almost all patients were characterized by the presence of at least two coexisting type 3 lesions (mean ± SD was 2.4 ± 1.1 for each patient using the rotational 3D visualization), the term “type 3 MNV complex” might be suggested as more suitable for referring to these lesions. Of note, both 2D and 3D OCTA images allowed for an increase detection of type 3 lesions in comparison with ICGA images. The latter results are not surprising assuming that the detection of these lesions may be challenging using ICGA. These difficulties may be secondary to the absence of hotspot in some post-nascent type 3 lesions and/or to the incapability of discriminate adjacent lesions.

Importantly, some of these identified lesions did not reach the sub-RPE space and suddenly interrupted within the outer retinal layers, as they might constitute immature (or nascent) lesions. The latter aspect reveals that different stages of type 3 NV may coexist. Moreover, some coexisting type 3 lesions were demonstrated to connect each other and merge into a single vascular trunk (e.g. case #3). Finally, even with this small cohort, two morphologic patterns could be easily discerned: “saccular”, and “filiform”.

Of note, the 3D image technique provides a way to assess the orientation of these lesions with respect to the retinal layers, RPE and Bruch’s membrane. In detail, each of these lesions appeared to have different orientations on the three dimensions and to move obliquely (forming angles >0 with the three axes of the three-dimensional Cartesian coordinate system) toward the outer retinal layers. Notably, these lesions seemed to become more perpendicular to the xy-plane, which was parallel to Bruch’s membrane (Fig. 2), near and under the RPE. We speculate that this orientation might be secondary to the presence of Müller cells, whose processes are known to have an oblique orientation within the retina and were demonstrated to guide the implant of type 3 lesions into the sub-RPE space^22–25^.

The main limitation of our study is the employment of a single time point for each patient. A prospective longitudinal evaluation of type 3 lesions will shed further light on the role of the three-dimensional approach in the follow-up of these lesions, and help to understand the clinical role of rotational 3D OCTA in the evaluation of treatment outcomes. Furthermore, it should be considered that rotational three-dimensional OCTA images must be interpreted with caution owing to a variety of artifacts. However, we used a novel prototype volume projection artifact removal algorithm to lighten this issue. Finally, another limitation is intrinsic to the OCTA device, which is not able to distinguish the absence of flow from that under the slowest detectable flow. Assuming this, we are not able to undoubtedly assert that those type 3 lesions interrupting in the outer retinal layers do not actually reach the sub-RPE space. Similarly, we are not able to exclude the absence of type 1 MNV in those cases not showing the presence of sub-RPE neovessels.

In conclusion, this study used a novel algorithm to obtain rotational three-dimensional visualization of treatment-naïve type 3 MNV. Our results are clinically relevant, as this approach seems to increase the detection performance of these lesions. In addition, it offered new insight into type 3 pathogenesis and characteristic. Rotational three-dimensional visualizations of treatment-naïve type 3 MNV may become a novel tool for monitoring the progression of type 3 macular neovascularizations.

## Supplementary information

Video Captions

Video 1

Video 2

## Data Availability

The data used to support the findings of this study are available from the corresponding author upon request.
