# Supplementary material for: Rotational Three-dimensional OCTA: a Notable New Imaging Tool to Characterize Type 3 Macular Neovascularization
Source: Sci Rep. 2019 Nov 19;9:17053. doi: 10.1038/s41598-019-53307-x (PMC6863896; doi:10.1038/s41598-019-53307-x)
Supplement: Supplementary file 1 — Video Captions [file 41598_2019_53307_MOESM1_ESM.pdf]

Supplementary information

Rotational Three-dimensional OCTA: a Notable New Imaging Tool to Characterize Type 3 Macular Neovascularization

Enrico Borrelli, MD, FEBO; Riccardo Sacconi, MD, FEBO;[1]Gerd Klose; Luis de Sisternes, PhD; Francesco Bandello, MD, FEBO; and Giuseppe Querques, MD, PhD.

VIDEO CAPTIONS

Video 1. Rotational 3D visualizations of three treatment-naïve type 3 MNV.

Video 2. Rotational 3D visualizations of the same type 3 NV before exudation and at the time of exudation ("post-nascent" stage).
